# Discovery of a haptoglobin glycopeptides biomarker panel for early diagnosis of hepatocellular carcinoma

**DOI:** 10.3389/fonc.2023.1213898

**Published:** 2023-10-18

**Authors:** Mahdokht Kohansal-Nodehi, Magdalena Swiatek-de Lange, Konstantin Kroeniger, Vinzent Rolny, Glòria Tabarés, Teerha Piratvisuth, Tawesak Tanwandee, Satawat Thongsawat, Wattana Sukeepaisarnjaroen, Juan Ignacio Esteban, Marta Bes, Bruno Köhler, Henry Lik-Yuen Chan, Holger Busskamp

**Affiliations:** ^1^ Roche Diagnostics GmbH, Research and Development Core Lab, Penzberg, Germany; ^2^ NKC Institute of Gastroenterology and Hepatology, Songklanagarind Hospital, Prince of Songkla University, Hat Yai, Thailand; ^3^ Division of Gastroenterology, Department of Medicine, Faculty of Medicine Siriraj Hospital, Mahidol University, Bangkok, Thailand; ^4^ Department of Internal Medicine, Maharaj Nakorn Chiang Mai Hospital, Chiang Mai University, Chiang Mai, Thailand; ^5^ Faculty of Medicine, Srinagarind Hospital, Khon Kaen University, Khon Kaen, Thailand; ^6^ Liver Unit, Hospital Universitari Vall d’Hebron (HUVH), Barcelona, Spain; ^7^ Transfusion Safety Laboratory, Banc de Sang i Teixits (BST), Barcelona, Spain; ^8^ Department of Medical Oncology, National Center for Tumor Diseases, University Hospital Heidelberg, Heidelberg, Germany; ^9^ Liver Cancer Center Heidelberg, Heidelberg, Germany; ^10^ Faculty of Medicine, The Chinese University of Hong Kong, Hong Kong, Hong Kong SAR, China

**Keywords:** haptoglobin, glycosylation, biomarker, glycoproteomics, hepatocellular carcinoma, diagnostics

## Abstract

**Background:**

There is a need for new serum biomarkers for early detection of hepatocellular carcinoma (HCC). Haptoglobin (Hp) N-glycosylation is altered in HCC, but the diagnostic value of site-specific Hp glycobiomarkers is rarely reported. We aimed to determine the site-specific glycosylation profile of Hp for early-stage HCC diagnosis.

**Method:**

Hp glycosylation was analyzed in the plasma of patients with liver diseases (n=57; controls), early-stage HCC (n=50) and late-stage HCC (n=32). Hp phenotype was determined by immunoblotting. Hp was immunoisolated and digested into peptides. N-glycopeptides were identified and quantified using liquid chromatography–mass spectrometry. Cohort samples were compared using Wilcoxon rank-sum (Mann-Whitney U) tests. Diagnostic performance was assessed using receiver operating characteristic (ROC) curves and area under curve (AUC).

**Results:**

Significantly higher fucosylation, branching and sialylation of Hp glycans, and expression of high-mannose glycans, was observed as disease progressed from cirrhosis to early- and late-stage HCC. Several glycopeptides demonstrated high values for early diagnosis of HCC, with an AUC of 93% (n=1), >80% (n=3), >75% (n=13) and >70% (n=11), compared with alpha-fetoprotein (AFP; AUC of 79%). The diagnostic performance of the identified biomarkers was only slightly affected by Hp phenotype.

**Conclusion:**

We identified a panel of Hp glycopeptides that are significantly differentially regulated in early- and late-stage HCC. Some glycobiomarkers exceeded the diagnostic value of AFP (the most commonly used biomarker for HCC diagnosis). Our findings provide evidence that glycobiomarkers can be effective in the diagnosis of early HCC – individually, as a panel of glycopeptides or combined with conventional serological biomarkers.

## 1 Introduction

Hepatocellular carcinoma (HCC) is the sixth most common cancer, and the fourth-leading cause of cancer-related death, worldwide (1). It is known that underlying liver diseases such as liver fibrosis and cirrhosis are the main risk factors for HCC (2). The 5-year survival rate of HCC patients is 70% upon early diagnosis; however, this rate decreases significantly to 15% when it is diagnosed in the late stages of the disease (3, 4). Unfortunately, due to lack of symptoms, more than 60% of HCC patients are diagnosed at a late stage, when metastasis has already occurred (5). Therefore, developing a non-invasive, early diagnostic test for HCC is essential to reduce the death rate and increase the efficiency of HCC treatment.

The most common methods for the diagnosis of HCC are ultrasound detection, imaging techniques such as computed axial tomography (CAT) scan or magnetic resonance imaging (MRI), and serological biomarkers. As shown previously, ultrasound detection has limited prognostic value, as it requires a tumor mass of at least 2 cm. Imaging techniques also have limitations such as poor sensitivity for early-stage tumors, operator dependency, and reduced quality in patients with obesity or non-alcoholic steatohepatitis (NASH). Therefore, in recent years, there has been an increased focus on the discovery of new serum biomarkers that can be used in surveillance programs for early detection of HCC in high-risk patients (6).

Alpha-fetoprotein (AFP) was one of the first serum biomarkers for HCC detection. However, it has shown suboptimal sensitivity and specificity for early detection of HCC (7, 8). It has also been reported that the AFP level might be falsely elevated in patients with chronic hepatitis or cirrhosis without HCC (9, 10). Protein induced by vitamin K absence/antagonist-II (PIVKA-II), also known as des-gamma carboxyprothrombin (DCP), is an abnormal form of prothrombin that is known to be elevated in HCC patients, and was therefore suggested as an alternative biomarker, or to be used in addition to AFP, for HCC detection.

In recent years, protein glycosylation analysis has gained a lot of attention as a method for discovering novel serological biomarkers and improving the clinical value of existing commonly used biomarkers (11). As an example, core fucosylation of AFP (AFP-L3) improved the diagnostic value of its protein counterpart, AFP. The diagnostic advantage of AFP-L3 is its high specificity to distinguish HCC from other liver diseases (12). In a phase 3 trial, the combination of AFP and AFP-L3 showed a sensitivity and specificity of 79% and 87%, respectively, for the detection of HCC in a cohort where 74% of patients were diagnosed with early-stage HCC (13). In a more recent attempt to increase the diagnostic value of serum biomarkers, researchers combined demographic risk factors such as age and gender with levels of AFP, AFP-L3 and PIVKA-II, to introduce a biomarker algorithm known as the GALAD score. Two studies (one from the US, and one from the UK, Germany and Japan), showed promising results with area under the curves (AUCs) of >89% for the diagnosis of early-stage HCC based on GALAD score; however, the algorithm is yet to be validated in larger cohorts and against control samples with a greater variety of demographic and etiological backgrounds (14, 15). Besides AFP, the hyper-fucosylation of other proteins such as Golgi-protein 73 (16), hemopexin (17), fetuin A (18), and ceruloplasmin (19), has been studied, and confirmed the overall increase in fucosylation of many proteins in patients with HCC (20).

Glycosylation pattern characteristics for NASH-related cirrhosis and HCC have been comprehensively studied by David M. Lubman et al. In particular, increased site-specific fucosylation, sialylation and isomeric glycan composition of haptoglobin differentiated better between cirrhosis and early NASH, with further improvements in AUC when combined with AFP compared with AFP alone (21, 22). The diagnostic potential of glycoproteins was further highlighted in a study assessing a state-of-the-art glycoproteomics approach for the identification of over 40 glycopeptides specific for NASH and its transition to HCC (23). Earlier, Goldman et al. identified 57 N-glycans that were significantly altered in HCC patients compared with chronic liver disease controls (24). A combination of three selected N-glycans was sufficient to classify HCC with 90% sensitivity and 89% specificity in an independent validation set of patients with chronic liver disease. Targeting abnormal glycosylation is also a potential therapeutic strategy for new HCC drugs. In this context, sorafenib, a kinase inhibitor drug approved for the treatment of advanced primary liver cancer, can inhibit the proliferation of cancer cells by altering their glycosylation status, including an increase in α-GalNAc, α-1,3GalNAc/Gal, β-1,3Gal, and GalNAcα-Ser/Thr(Tn) structures, and a decrease in GlcNAc, tetra-antennary complex-type N-glycan, sialic acid, and β-1,4Gal structures (25).

In the present study, we focused on haptoglobin (Hp), an acute-phase glycoprotein, with four N-glycosylation sites, as the target for discovering new early-stage HCC biomarkers. As with most serum tumor biomarkers, Hp undergoes aberrant glycosylation modifications and is thought to play a vital role in alteration of the extracellular matrix, formation of the tumor microenvironment and regulation of the immune response in HCC through N-glycosylation (26). Several comprehensive studies have already reported an elevated level of fucosylated and sialylated Hp in patients with early-stage HCC compared with patients with cirrhosis in both viral- and non-viral (NASH) etiologies (21, 22, 27–31).

Here, we sought to conduct an in-depth site-specific characterization of Hp glycan composition in a cohort of patients with a variety of liver diseases, and early- and late-stage HCC, with the aim of reinforcing previous findings and to potentially reveal other unique glycopeptide structures with high value for the diagnosis of early-stage HCC.

## 2 Materials and methods

### 2.1 Human serum and plasma samples

Samples, including ethylenediaminetetraacetic acid (EDTA)-plasma and sera were collected from patients with different liver diseases such as chronic hepatitis B and C, hepatic fibrosis, NASH and alcoholic steatohepatitis (control group), and from patients in the early and late stages of HCC. The samples were provided by the following institutions: Maharaj Nakorn Chiang Mai Hospital, Chiang Mai, Thailand; Siriraj Hospital, Bangkok, Thailand; Songklanagarind Hospital, HatYai Songkhla, Thailand; Srinagarind Hospital, KhonKaen, Thailand; Prince of Wales Hospital, Shatin, Hong Kong; NCT Universitätsklinikum Heidelberg, Heidelberg, Germany; and University Hospital Vall d’Hebron, Barcelona, Spain. Informed written consent was obtained from all participating patients. The study was conducted in full conformance with the principles of the Declaration of Helsinki and with approval from the Independent Ethics Committee (IEC).

Samples were collected before treatment (surgery, percutaneous ethanol injection (PEI), chemotherapy, or radiotherapy) according to the appropriate standard operating procedures and stored at <-70°C until analysis. Repeated freeze–thawing was avoided. HCC diagnosis was verified using imaging (sonography, computed tomography [CT], magnetic resonance imaging [MRI]) or biopsy, followed by histopathological analysis. The stage classification of samples was based on the Barcelona Liver Cancer (BCLC) guidelines (32), as endorsed by the American (AASLD) and European (EASL) Associations for the Study of Liver/Liver Diseases (33, 34). In this staging system, HCC disease progression is divided into five stages (0=very early, A=early, B=intermediate, C=advanced, D=terminal) based on the number and size of the tumor nodes (32). In our study we combined stages 0 and A as the early-stage HCC group and stages B, C, and D as the late-stage HCC group.

### 2.2 Measurement of AFP concentration

AFP was measured in serum samples using microchip capillary electrophoresis and a liquid-phase binding assay on the uTASWako i30 automated analyzer (Fujifilm Wako Pure Chemical Industries, Osaka, Japan).

### 2.3 Hp phenotype analysis – immunoblotting

The Hp phenotype of sera samples was characterized by immunoblotting using an antibody against the Hp α-chains (Abcam; Waltham, USA). Briefly, 2 µl of EDTA-plasma were diluted (10 times) with sample buffer (2X) and dithiothreitol (DTT) (25 mM) and heated for 10 min at 70°C. Suzuamples were cooled at room temperature (RT), before 10 µl of each were loaded onto an SDS-PAGE gel. The proteins were separated based on molecular size and transferred to a polyvinylidene difluoride (PVDF) membrane for immunoblotting with a primary antibody against Hp α-chains, followed by horseradish peroxidase (HRP)-conjugated mouse antibody. Finally, the membrane was developed with an enhanced chemiluminescence reagent, and signals from the immunoreactive proteins were detected with a LAS-1000 imaging system. The exclusive presence of α1- and α2-chains characterized the Hp phenotype as type 1-1 and type 2-2, respectively. The presence of both α1- and α2-chains was defined as type 2-1.

### 2.4 Hp immunoisolation – immunoprecipitation

Hp was immunoisolated from EDTA-plasma using antibody-coupled beads: 150 µg of an in-house biotin-labeled F(ab’)2 fragment against the Hp β-chain was coupled to 10 mg of streptavidin beads (1/≈66.6 of antibodies/beads ratio) on a rotator for 1 hour at RT; beads were then washed three times with phosphate-buffered saline (PBS) buffer (10 mM phosphate buffer, 2.7 mM KCl, 137 mM NaCl, pH: 7.4). Fifty microliters of plasma were incubated with 5 µl of 20 mM DTT for 1 hour at 37°C, and then treated with 10 µl of 50 mM iodoacetamide (IAM) for 30 min at RT. The samples were diluted with 2 ml PBS and incubated together with the antibody-coupled beads from the previous step. After a 2-hour incubation at RT on a rotator, unbound proteins were washed with two steps of PBS buffer+0.1% Tween 20 (PBST) and two steps of PBS buffer. The bound Hp was then eluted from the beads with 500 µl of glycine (0.2 M) buffer (pH: 2.6) in two steps. To neutralize the pH of the eluted samples, 200 µl NaOH (1 N) was added to the eluted fraction after each elution step.

### 2.5 Filter aided sample preparation (FASP) digestion

The eluted Hp fraction from the previous step was loaded on the Nanosep filter (cut off: 10 kDa) and centrifuged at 10,000 g for 20 min. Then, 75 ng of heavy Hp protein (recombinant Hp with isotopically labeled heavy Lys and Arg) was added to each sample as an internal standard. After this, 50 µl of denaturing buffer (1 mg/ml protein precipitation solution (3-[3-(1,1-bisheptyloxyethyl)yridine-1-yl]propan-1-sulfonat) in 50 mM ammonium bicarbonate (ABC) and 5 µl of DTT (10 mM) were added to the samples and incubated at 50°C for 30 min. In the next step, 5 µl of IAM (55 mM) was added to the samples, followed by an incubation for 30 min at 37°C in the dark. Samples were then centrifuged at 10,000 g for 20 min to wash out the buffer and washed once with 100 µl of ABC buffer. Proteins were digested on the filter by addition of 6 µg of trypsin in 50 µl of ABC buffer to each sample for 3 hours at 37°C. Digestion was stopped by incubating at 95°C for 10 min. For the second digestion, 10 µg of Glu-C was applied for each sample and incubated overnight at 25°C. The next day, digested samples were eluted from the membrane by one-step centrifugation at 10,000 g for 20 min.

### 2.6 Liquid chromatography–mass spectrometry (LC-MS)/MS analysis

For further analysis, 20 µl of the digested peptides were injected into the LC-MS/MS system (HF-X mass spectrometer, Thermo Fisher Scientific, Germany) coupled to the Vanquish ultra-high-performance liquid chromatography (UHPLC) system (Thermo Fisher Scientific, Germany). The peptides were separated by reverse phase chromatography on a C18 column (Waters, XSelect CSH C18 Column, 130Å, 3.5 µm, 2.1 mm X 150 mm), incubated at 50°C. The LC gradient was set at 320 µl/min flow rate as follows: 0%−30% B (0–30 min), 30%−80% B (30–31 min), 80% B (31–36 min), 80%–0% B (36–37 min), and 0% B (37–42 min), where the eluent A and B were H_2_O containing 0.1% formic acid, and acetonitrile containing 0.1% formic acid, respectively. The separated peptides were ionized by an electrospray ionization (ESI) source and analyzed in the positive mode using the data-dependent acquisition method. Full scan MS spectra were acquired in the range of 300–2000 m/z at a resolution of 60,000, 10e6 automatic gain control (AGC), and 50 MS injection time. The five most intense peaks from the survey scan were selected for fragmentation with higher energy collisional dissociation (HCD), at the normalized collision energy of 28, resolution of 15000, 1e5 automatic gain control (AGC), and 150 ms injection time. In order to monitor the reproducibility of the measurement over time, a control sample comprising the mixture of all cohort samples was prepared using the same protocol as the cohort samples and measured during the 22 days of MS measurement.

### 2.7 Data analysis

**Figure 1:**
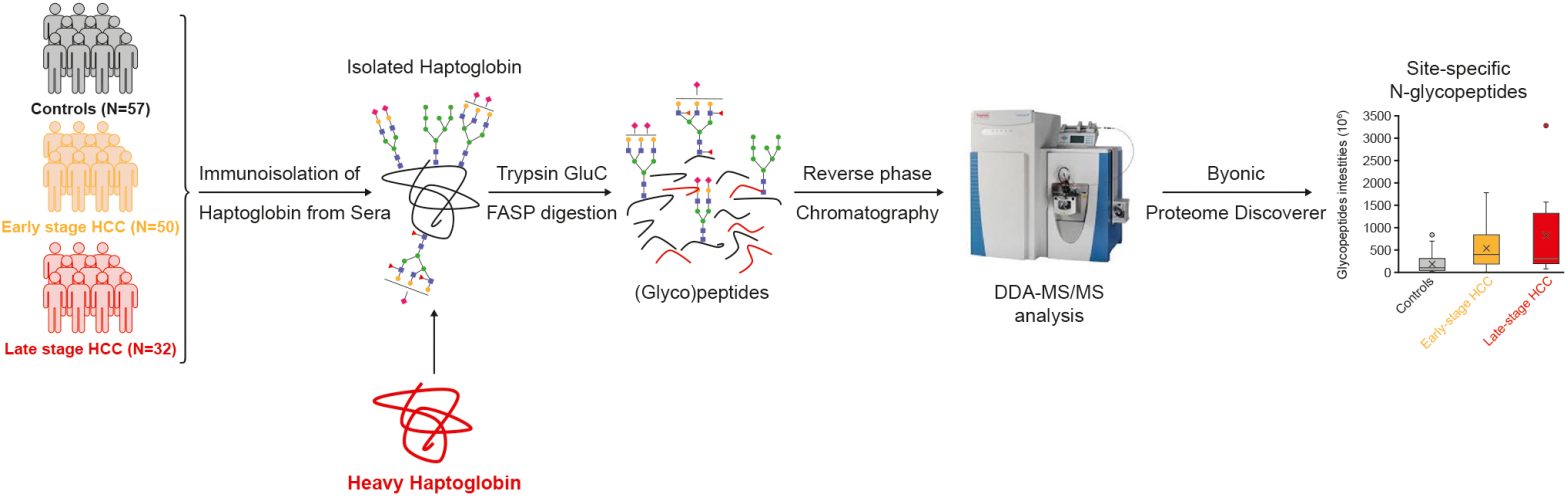
Study design and workflow. Haptoglobin was immunoisolated from the sera of a cohort of controls, early and late-stage HCC patients. The heavy Hp was added to samples as internal standard. Isolated Hp was denatured, reduced, alkylated, and digested into peptides by FASP and measured by LC-MS/MS. Byonic and Proteome Discoverer softwares were used for identification and quantification of glycopeptides. DDA, data-dependent acquisition; FASP, filter aided sample preparation; HCC, hepatocellular carcinoma; LC, Liquid chromatography; MS, mass spectrometry.

The acquired raw files from the mass spectrometer were processed by the Byonic search engine (Protein Metrics, CA, US) embedded in Proteome Discoverer 2.2 (Thermo Fisher Scientific). The dataset was searched against the UniProt Hp protein sequence (P00738). To identify glycopeptides, Byonic-curated databases (309 mammalian N-glycans with no sodium, 57 human plasma N-glycans and 182 human N-glycans without multiple fucose, combined in one search) were used. The Byonic Proteome Discoverer was configured as follows: mass tolerance was set to 10 ppm for MS1 and 20 ppm for MS2. Glu-C and trypsin were set as proteases, allowing for two missed cleavages; carbamidomethylation of cysteine was set as fixed modification, and methionine oxidation and glycosylation on asparagine were specified as variable modifications. The results were filtered at a 1% false discovery rate (FDR) and with a confidence threshold of the Byonic score >100. The composition of glycopeptides with significant differences between cohort groups and with AUC >70% were manually reviewed – this involved checking retention time, charge state, glycan oxonium ions and monoisotopic peaks. In some cases, identification of the glycopeptide could not be restricted to one glycan composition after the manual check, due to the presence of diagnostic peaks of core- or antennary-fucose as well as sialic acid oxonium ions (e.g. in the case of 2Fuc-1.02 = 1NeuAc). In such cases, all possible compositions that matched the m/z of the glycopeptide are mentioned in the text/figures. In the text, glycan composition is referred to as follows: HexNAc(x)Hex(x)Fuc(x)NeuAc(x), where HexNAc, Hex, Fuc, and NeuAc represent N-acetylglucos(galactose)amine, mannose (glucose, galactose), fucose and N-acetylneuraminic acid, respectively. The numbers in parentheses represent the number of monomers in the glycans. In the text, ‘glyco-compounds’ refers to the combination of peptide (glycosite) and glycan with distinctive m/z. This might include two different glycans that have different structures or composition but can be annotated to one m/z. Schematic representation is according to the Symbol Nomenclature For Glycans (SNFG) hosted by the National Center for Biotechnology Information (NCBI) (35). Peak areas of glycopeptides extracted ion chromatograms (XICs) were automatically integrated by the Proteome Discoverer and used as relative quantitative values. The identification and quantification of glycopeptides is shown in **Figure 1**.

### 2.8 Statistical analysis

To correct for possible digestion or MS measurement variations, the abundance of the glycopeptides in the samples was normalized to the abundance of the top three peptides of the spiked-in heavy Hp that was added after immunoprecipitation and before digestion to each sample. Subsequently, the missing values were replaced by the minimum abundance value of that glycopeptide in the dataset. To test the significant differences in glycopeptides between the clinical groups, p-values were calculated using Wilcoxon rank-sum (Mann-Whitney U) tests. In order to correct for multiple testing, the Benjamini-Hochberg procedure, with an FDR control of 20% was used. To evaluate the diagnostic performance of significant glycopeptides, receiver-operating characteristic (ROC) curves were constructed and area under the curve (AUC) values were calculated using the pROC package (v.1.15.3) within the R software (v3.5.2; available at https://www.R-project.org) (36–39). Specificity values were calculated at a fixed value of 90% sensitivity, and sensitivity values at 90% specificity.

## 3 Results

The developed workflow (described in the Methods) was used to analyze samples from 57 patients with liver diseases (control group), 50 patients in early-stage HCC, and 32 patients in late-stage HCC. Detailed patient demographics are summarized in **Table 1**.

**Table 1:** Patient characteristics in the clinical evaluation cohort.

Characteristics	Early-stage HCC (N=50)	Late-stage HCC (N=32)	All-stage HCC (N=82)	Control (N=57)	Total (N=139)
Age					
Mean	61	61	61	54	58
SD	8.8	10	9.4	10	10
Median	61	60	61	54	58
P25–P75	57–66	52–70	55–68	48–60	52–65
Min–Max	38–78	44–79	38–79	29–80	29–80
Missing, %	0 (0.0%)	0 (0.0%)	0 (0.0%)	0 (0.0%)	0 (0.0%)
N	50	32	82	57	139
Sex					
Female	11 (22.0%)	2 (6.3%)	13 (16.0%)	23 (40.0%)	36 (26.0%)
Male	39 (78.0%)	30 (94.0%)	69 (84.0%)	34 (60.0%)	103 (74.0%)
Type					
Type 1-1	4 (8.0%)	2 (6.3%)	6 (7.3%)	3 (5.3%)	9 (6.5%)
Type 1-2	16 (32.0%)	15 (47.0%)	31 (38.0%)	33 (58.0%)	64 (46.0%)
Type 2-2	26 (52.0%)	12 (38.0%)	38 (46.0%)	20 (35.0%)	58 (42.0%)
Type-Not identified	4 (8.0%)	3 (9.4%)	7 (8.5%)	1 (1.8%)	8 (5.8%)
Etiology					
Alcoholic steatohepatitis (with or without cirrhosis)	4 (8.0%)	5 (15.6%)	9 (11.0%)	0 (0.0%)	9 (6.5%)
Alcoholic steatohepatitis with chronic hepatitis B	3 (6.0%)	1 (3.1%)	4 (4.9%)	1 (1.8%)	5 (3.6%)
Alcoholic steatohepatitis with chronic hepatitis C	4 (8.0%)	0 (0.0%)	4 (4.9%)	1 (1.8%)	5 (3.6%)
Non-alcoholic steatohepatitis	1 (2.0%)	1 (3.1%)	2 (2.4%)	0 (0.0%)	2 (1.4%)
Non-alcoholic steatohepatitis with chronic hepatitis B	0 (0.0%)	1 (3.1%)	1 (1.2%)	0 (0.0%)	1 (0.7%)
Chronic hepatitis B (with or without cirrhosis)	17 (34.0%)	13 (40.6%)	30 (36.6%)	42 (73.7%)	72 (51.8%)
Chronic hepatitis C (with or without cirrhosis)	13 (26.0%)	7 (21.8%)	20 (24.4%)	13 (22.8%)	33 (23.7%)
Chronic hepatitis B and chronic hepatitis C, hepatic cirrhosis	0 (0.0%)	1 (3.1%)	1 (1.2%)	0 (0.0%)	1 (0.7%)
Hepatic cirrhosis	6 (12.0%)	3 (9.4%)	9 (11.0%)	0 (0.0%)	9 (6.5%)
Hepatic cirrhosis, other	0 (0.0%)	1 (3.1%)	1 (1.2%)	0 (0.0%)	1 (0.7%)

HCC, hepatocellular carcinoma; SD, standard deviation.

A feasibility study demonstrated that the pattern of glycopeptides isolated from native EDTA-plasma does not differ from the pattern generated from matched serum (data not shown).

There was a high reproducibility of measurement over the 22 days of measurement with a control sample (pool of all cohort samples), as demonstrated by a mean coefficient of variation (CV) of 10.66% (range: 8.09%–12.83%) on the Hp protein level and 8.79% (range: 6.61%–9.86%) on the top three peptides level.

### 3.1 Site-specific microheterogeneity and dynamic range of plasma Hp glycoforms

**Figure 2:**
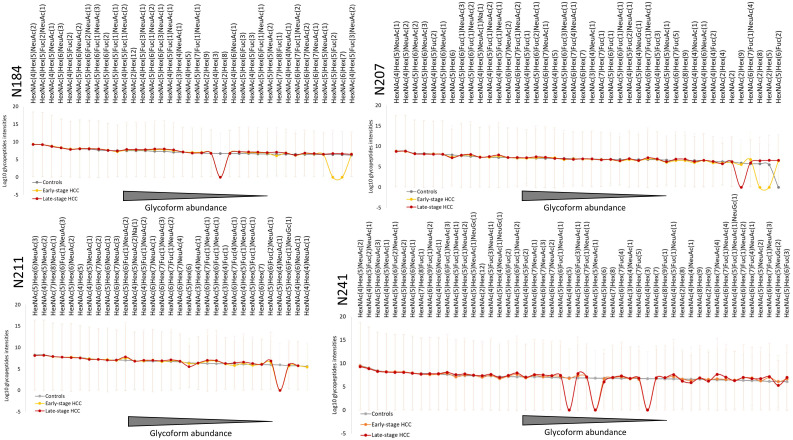
Overview of Haptoglobin glycosylation pattern in all four glycosites. The average intensities of quantified Hp glycoform compositions in each group are depicted in order of abundance for each glycosite. Combining all the three conditions, 36, 42, 31 and 50 glycoforms were identified at N184, N207, N211, N241, respectively. In all four glycosites, the overall pattern of glycoforms’ abundance in early- and late-stage HCC are similar to each other but different compared with cirrhosis. The highly abundant glycoforms show less alteration between cirrhosis and HCC (early- or late-stage), whereas most of the differences can be observed in the intermediate- and low-abundant glycoforms. The abundance of not identified glycoforms in each group are considered as 0 (in Log10 scale shown here as 1). HexNAc, Hex, Fuc, and NeuAc represent N-acetylglucos(galactose)amine, Mannose (Glucose, Galactose), Fucose and N-acetylneuraminic acid, respectively. HCC, hepatocellular carcinoma; Hp, haptoglobin.

In total, 170, 132 and 165 site-specific glycopeptides were identified in controls and early- and late-stage HCC samples for all four glycosites on the Hp β-chain, respectively. Among them, 36 glycoforms were identified at N184, 42 at N207, 31 at N211 and 50 at N241. This is in line with previous studies, showing highest glycosylation microheterogeneity at the N241 site (22, 29, 31). Most of the identified glycopeptides contained complex glycans containing fucose and sialic acid. High-mannose glycans were also identified at three of the four glycosites, which has rarely been reported in previous studies. In addition, a high dynamic range (10^5^) was observed between the most and the least abundant glycopeptides at all glycosites. Considering all disease conditions and glycosites, the most abundant glycopeptides contained biantennary glycans with one or two sialic acids and without any fucose (HexNAc(4)Hex(5)NeuAc(1) or HexNAc(4)Hex(5)NeuAc(2)). Significant differences in the expression of the highly abundant glycoforms were rarely observed between the three disease conditions on all four glycosites (**Figure 2**). Conversely, some low-abundant glycoforms were not identified in one or more conditions (intensity=0, **Figure 2**). This might be due to the fact that these glycoforms were completely absent from the sample or being so low in abundance that they were not measurable by our method. Overall, the abundance of glycoforms were very similar in early- and late-stage HCC but varied in the control group. In most cases, the alterations of glycosylation in early-stage HCC (compared with controls) were milder than for late-stage HCC. As the HCC progressed from early- to late-stage, more intense changes in the abundance of glycostructures were observed (**Figure 2**).

### 3.2 Upregulation of glycans with specific characteristics in HCC

**Figure 3:**
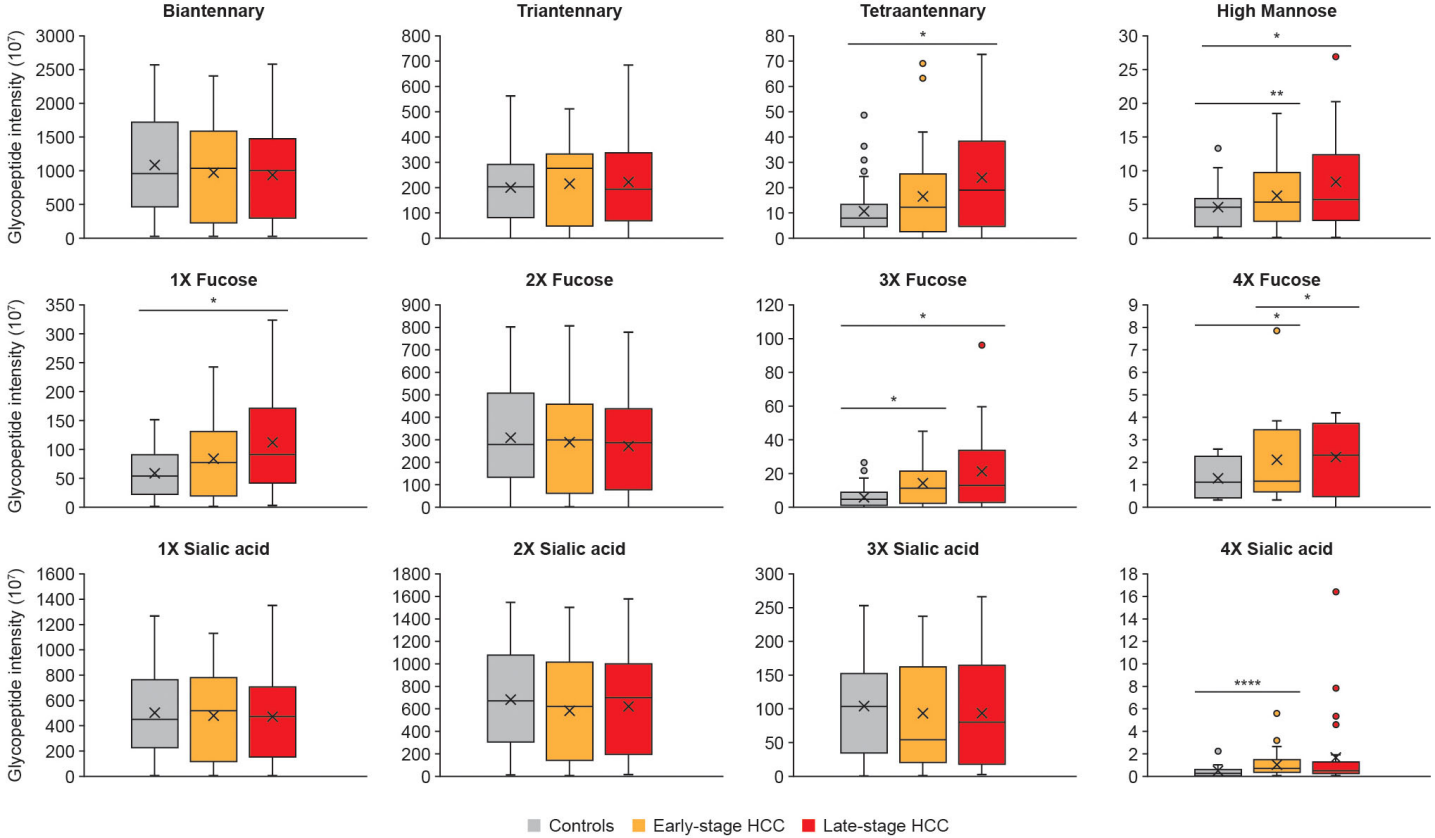
Specific glycan characteristics upregulated in HCC. The abundance of glycopeptides with similar characteristics such as branching, fucosylation and sialylation were summed up and compared between controls, early- and late-stage HCC cohorts. We observed tetraantennary and monofucosylation significantly increased in late-stage compared with cirrhosis; whereas the tri- and tetrafucosylation and high mannose glycans increased as HCC progressed from cirrhosis to early- and then late-stage HCC. Significant differences in sialylation were only detected for tetrasialyation, which was increased in early-stage HCC compared with controls. Statistically significant differences were determined by using the Wilcoxon rank-sum (Mann-Whitney U) tests. The *, **, **** represent p-values lower than 0.05, 0.01 and 0.0001, respectively. Standard deviations for each panel are shown as error bars.

Following the identification and quantification of glycopeptides in the cohort, the upregulated glycoforms were evaluated for any unique characteristics correlating with HCC. To this end, the intensity of glycans presenting 1-4 fucoses, 1-4 sialic acids, branches (bi-, tri-, and tetra-antennary) or high-mannose glycans were summed, and then checked for significant alterations between controls, and early- and late-stage HCC. The data showed that tetra-antennary glycans were expressed higher in both early- and late-stage HCC compared with controls, however, the difference was only significant in late-stage HCC. Interestingly, high-mannose glycans were significantly increased in both early- and late-stage HCC compared with controls (**Figure 3**).

The most significant differences were in fucosylated glycans. Mono-, tri, and tetra-fucosylated glycans were increased in late-stage HCC, and tri-, and tetra-fucosylated glycans were increased in early-stage HCC, compared with controls (**Figure 3**). Tetra-sialylation was also significantly increased in early-stage HCC when compared with controls (**Figure 3**).

### 3.3 Clinical significance of site-specific glycopeptide analysis for early diagnosis of HCC

**Figure 4:**
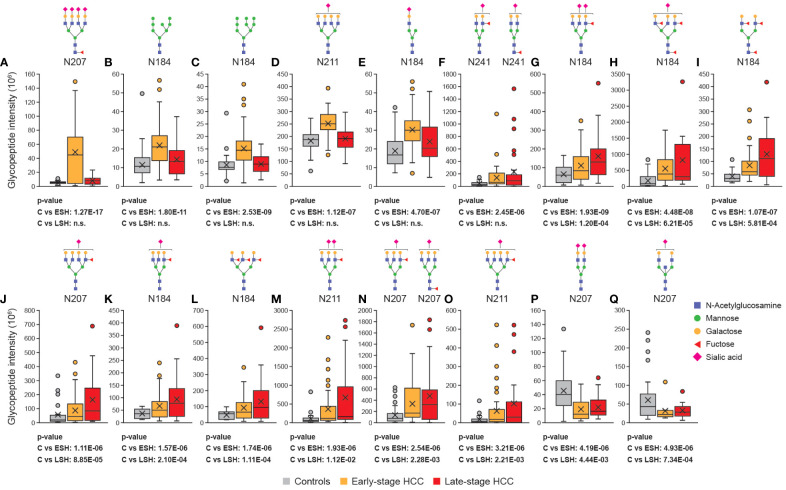
Site-specific glycan analysis revealed differentially regulated glycopeptides in HCC. The abundance of Hp glycopeptides (with putative N-glycan structures) were compared between controls, early- and late-stage cohorts. Some of the glycopeptides were specifically upregulated in early-stage HCC **(A–F)**, some were upregulated both at the early- and late-stage HCC **(G–O)** and some of them were downregulated in early- and late-stage HCC compared with cirrhosis **(P, Q)**. All the glycan compositions that can be matched to an m/z are depicted in case of ambiguous identification. Statistically significant differences between cirrhosis, early-stage HCC and late-stage HCC were determined using the Wilcoxon rank-sum (Mann-Whitney U) tests. C, cirrhosis; ESH, early-stage HCC; HCC, hepatocellular carcinoma; Hp, haptoglobin; LSH, late-stage HCC; HCC, hepatocellular carcinoma.

The overall analysis of glycan characteristics (e.g. fucosylation) provides insight into major changes in the glycosylation pattern of Hp upon HCC progression. However, since the types and abundance of glycoforms vary at different Hp glycosites (see results above), it was hypothesized that Hp site- and glycoform-specific analyses would reveal detailed and pronounced changes in glycosylation that correlate with HCC progression. The analyses showed that certain glycopeptides were significantly upregulated in the early stages of HCC, including glycopeptides that are highly branched (**Figures 4A, D**), mono-branched (**Figure 4E**), fucosylated (**Figures 4A, F**), and highly mannosylated (**Figures 4B, C**). Some of the glycopeptides identified were attributed to multiple glycans because the MS2 spectra of the glycopeptides had evidence for more than one suggested glycan structure (**Figure 4F**). In other cases, most likely structure could be determined after additional data analysis, for example, for the highest upregulated N207 glycopeptide in early-stage HCC (compared with controls; **Figure 4A**) two glycan compositions can be proposed: HexNAc(6)Hex(7)Fuc(1)NeuAc(4) and HexNAc(6)Hex(7)Fuc(3)NeuAc(3). The monoisotopic peak and diagnostic oxonium ions of those glycopeptides were checked, and showed sialic acid oxonium ions (e.g. 274.0919) and both the core-(Hex+HexNAc+Fuc, m/z 512.20) and outer-arm (pep+2HexNAc+Fuc, m/z 1524.7457) fucose diagnostic peaks. Since the abundance of outer-arm fucose oxonium ions (m/z 1524.7457) was very low and was not present in all the samples, it was concluded that the HexNAc(6)Hex(7)Fuc(1)NeuAc(4) structure was dominant.

Besides early-stage HCC-specific biomarkers, nine other glycopeptides (HexNAc(5)Hex(6)Fuc(1)NeuAc(2), HexNAc(5)Hex(6)Fuc(3)NeuAc(1), HexNAc(4)Hex(5)Fuc(3), HexNAc(6)Hex(7)Fuc(1)NeuAc(1), HexNAc(5)Hex(6)Fuc(1)NeuAc(1), HexNAc(5)Hex(6)Fuc(3), HexNAc(5)Hex(6)Fuc(1)NeuAc(2), two possible HexNAc(5)Hex(6)Fuc(1)NeuAc(1) structures that cannot be differentiated, and HexNAc(6)Hex(7)Fuc(1)NeuAc(1)), were significantly overexpressed in both early- and late-stage HCC (**Figures 4G–O**), with expression increasing from early- to late-stage disease. Interestingly, all of these glycan structures were highly branched (tri- or tetra-antennary) and had at least one fucose in their structure.

In addition to upregulated glycopeptides in HCC, two glycopeptides – located at the N207 and N241 glycosites – were significantly downregulated in early- and late-stage HCC compared with controls (**Figures 4P, Q**). One of the glycoforms was a biantennary glycan with two sialic acids (HexNAc(4)Hex(5)NeuAc(2); **Figure 4P**), which is among the most abundant glycans, and the other glycoform was a bisected biantennary glycan with one sialic acid (HexNAc(5)Hex(5)NeuAc(1); **Figure 4Q**). Overall, this observation confirmed the hypothesis that upon the progression from cirrhosis to HCC, the less branched and decorated Hp glycoforms are converted to highly branched and fucosylated glycoforms.

**Figure 5:**
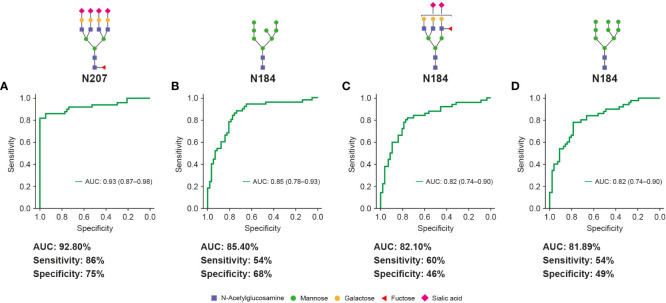
Unique Hp glycopeptides demonstrate high clinical values for early-stage HCC diagnosis. Glycopeptides (with putative N-glycan structures) that are highly fucosylated and sialylated **(A, C)** or contain high-mannose glycans **(B, D)** showed high clinical values (AUC, sensitivity and specificity) for diagnosis of early-stage HCC and distinguish it from cirrhosis. The specificity and sensitivity values are reported at fixed sensitivity and specificity cutoffs of 0.9, respectively. AUC, area under curve; HCC, hepatocellular carcinoma.

ROC curves were generated to evaluate the diagnostic potential of significantly up- and down-regulated glycopeptides as early-stage HCC biomarkers. ROC analysis revealed one glyco-compound with an AUC of 92.81% (sensitivity: 86%, specificity: 75%; **Figure 5A**), three glyco-compounds with AUC >80% (**Figures 5B–D**), 13 glyco-compounds with AUC >75% (**Supplementary Figure 1**), and 12 further glyco-compounds with AUC >70% (data not shown). Most importantly, some of the potential glycopeptide biomarkers described above exceeded the AUC of AFP (79%) and thus also improved the clinical value of AFP, as the commonly accepted HCC biomarker for the same cohort (**Supplementary Figure 2**).

### 3.4 Effect of HCC etiologies on the diagnostic performance of glycobiomarkers

As major risk factors for developing cirrhosis and HCC are infections with hepatitis B (HBV), hepatitis C (HCV) and alcoholic steatohepatitis, we attempted to evaluate best performing Hp glycoforms in specific HCC etiology groups with at least 10 patients per group. As there were a number of patients diagnosed with more than one etiology, and patient numbers for some etiologies or stages were below 10, the analyses could only be performed for patients of viral etiologies: HBV (N=28 [n=14 early-stage HCC; n=14 late-stage HCC]) and HCV patients (N=20 [n=16 early-stage HCC; n=4 late-stage HCC]) (**Supplementary Table 1**). In addition, we analyzed the performance of glycopeptides in cirrhosis (N=66 [n=40 early-stage HCC; n=26 late-stage HCC]) and non-cirrhosis groups (N=16 [n=10 early-stage HCC; n=6 late-stage HCC]) independent of etiologies (**Supplementary Table 1**).

For the detection of early-stage HCC, glycopeptide HexNAc(6)Hex(7)Fuc(1)NeuAc(4) at position N207 showed reproducibly good performance in all examined etiologies: cirrhotic HBV (AUC 86.3), HCV (AUC 93.3%), all cirrhotic (AUC 92.5%) and non-cirrhotic patients (AUC 196.5.0%) (**Supplementary Table 2**). A similar effect was observed for glycan HexNAc(2)Hex(8) at position N184, with AUC being 75.0%, 74.6%, 78.3% and 87.4% for cirrhotic HBV, HCV, all cirrhotic, and all non-cirrhotic patients, respectively. Stable performance of the HexNAc(5)Hex(6)Fuc(1)NeuAc(2) glycopeptide at position N184 (indicative for both early- and late-stage HCC) was also observed across all etiologies (**Supplementary Table 2**). AUCs for AFP were 68.5%, 61.2%, 63.2% and 89.4% for cirrhotic HBV, HCV, all cirrhotic, and all non-cirrhotic patients, respectively.

### 3.5 Effect of Hp phenotype on the diagnostic performance of glycobiomarkers

**Figure 6:**
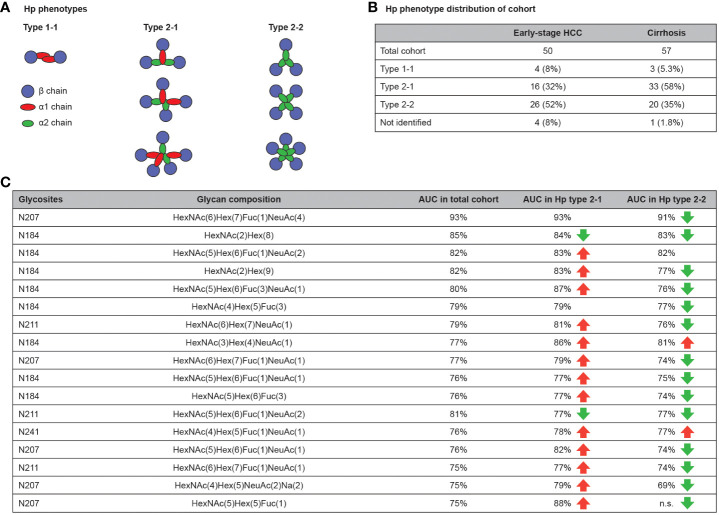
Hp phenotype distribution affects the clinical value of Hp glycopeptides. Hp have three different phenotypes in the population that are defined by the type of Hp α chain (α1 and α2) and the number of polymers that they form in blood **(A)**. Hp phenotype distribution of used cohort **(B)**. AUC comparison of glycopeptides for diagnosis of early-stage HCC in total cohort compared with Hp type 2-1 and 2-2. Beside some exceptions, the AUC slightly increased in the type 2-1 subset and slightly decreased in type 2-2 subset compared with the total cohort. **(C)** AUC, area under curve; HCC, hepatocellular carcinoma; Hp, haptoglobin.

Two common alleles of Hp exist in the population, Hp1 and Hp2, and the presence of an α1 chain, an α2 chain or both, determines the phenotype of Hp as type 1-1, 2-2 or 2-1, respectively (**Figure 6A**) (40). Therefore, it was hypothesized that the baseline difference in glycosylation pattern of Hp phenotypes could affect their diagnostic value. To test this hypothesis, the Hp type of the cohort samples were characterized using denaturing protein gel electrophoresis (SDS-PAGE) followed by specific immunoblotting. Among the early-stage HCC samples, four (8%), 16 (32%) and 26 (52%) patients were characterized as type 1-1, 2-1 or 2-2; among disease control samples three (5.3%), 33 (58%) and 20 (35%) patients were characterized as type 1-1, 2-1 or 2-2, respectively (**Figure 6B**). This percentage distribution of phenotypes is in line with the Western European population (41). In the next step, the AUC of glycopeptides was calculated separately in Hp 2-1 and 2-2 (type 1-1 was excluded due to the low number of samples) and compared with the AUC of the same glycopeptides in the total cohort of samples. Overall, there was a slight increase in AUC values among type 2-1, and a small decrease among type 2-2, compared with the AUC in samples from the total cohort (**Figure 6C**). However, this variation was minor in the most significant glycobiomarkers (AUC >80%) and did not affect their performance.

## 4 Discussion

It is known that underlying liver conditions such as viral hepatitis, non-alcoholic fatty liver, NASH, fibrosis and cirrhosis are the main risk factors for HCC. A range of genetic and environmental modifiers define the probability of progression from these liver diseases to HCC (42), which creates an urgent need to discover and validate new biomarkers that distinguish between chronic liver diseases and the early stages of HCC. Early diagnosis of HCC would allow medical intervention with early-line HCC treatments such as liver ablation or transplantation, to improve patient survival.

In this study, we investigated the value of alterated glycosylation of Hp as a diagnostic glycobiomarker for early detection of HCC in a panel of patients with liver diseases (as controls), and in cases of early- and late-stage HCC.

There are numerous previous studies aiming to identify Hp glycobiomarkers for HCC, but using different approaches. An alternative workflow was applied for only released glycans (no information regarding glycan site-specificity in Hp protein) (27, 28, 43), or focused on the selected glycosites, e.g. only N184 and N241 (22, 31). A common shortcoming of these studies was also the low number of analyzed samples (29, 30). Thus, we designed our study to address these issues by evaluating a larger cohort, and we applied site-specific glycoproteomics methods in order to characterize the alteration in glycan composition at each Hp glycosite separately as the condition progresses from various liver diseases to early- and late-stage HCC.

Based on our results, we observed several clinically relevant points: *(1)* Hp fucosylation is significantly higher in both early- and late-stage HCC compared with controls, which agrees with previous reports using lectin- or mass spectrometry-based methods showing high fucosylation of Hp not only in HCC, but also in other cancers (23, 28, 44, 45). This could be explained by the overexpression of enzymes that are involved in protein fucosylation (e.g. FUT8) or of their substrates (46–48). Another explanation may be that cancer cells lose their polarity and therefore secrete fucosylated proteins into circulation that are not normally secreted (49). *(2)* We observed an increase of tetra-antennary glycans in early-HCC and a significant increase in late-stage HCC compared with controls. This phenomenon has been reported before and it is assumed that the main reason is overexpression of the MGAT-V gene, which is responsible for the addition of β-1,6-GlcNAc to growing N-linked glycans, converting them to tri- and tetra-antennary glycan polymers (50, 51). *(3)* High-mannose glycans showed a significant increase in early- and late-stage HCC compared with controls, which to our knowledge, is a novel observation.

Increase of high-mannose glycans has been reported before in breast (52), lung (53) and colorectal (54) cancers. Also, a previous study demonstrated an increase of high-mannose glycans in HCC using a lectin microarray method (55). It is hypothesized that as the cancer cells go through the de-differentiation process, they express more high-mannose glycans, which are normally used as precursors for more complex glycans. Therefore, an increase in high-mannose glycans may be associated with a high-grade HCC malignancy (55). Overall, our study revealed specific glycan characteristics such as branching, fucosylation and high-mannosylation to be upregulated in early- and late-stage HCC, which is especially important for designing tests based on specific glycan binders such as lectins or antibodies.

Another important clinical implication of the current study is the discovery of a new panel of glycopeptides as biomarkers for early-stage HCC (one glycopeptide with an AUC of >90%, three with AUC >80% and 13 with AUC >75%), which are able to improve on the diagnostic values of commonly used biomarkers, such as AFP. This panel of glycobiomarkers could be translated into diagnostic tests using glyco-specific binders or programmed targeted LC-MS/MS methods. In addition, previous studies on HCC glycobiomarkers have shown that they could be used for the prognosis of HCC, 18 months in advance (56). This suggests that glycosylation patterns of Hp in HCC patients may start to change long before any tumor node can be detected by conventional methods. Interestingly, in their recently published work, Ramachandran et al. (23) identified a very similar set of glycans to those highlighted in our study. However, in most cases, these structures were not attached to Hp, but rather to other abundant glycoproteins including α-1-antitrypsin, α-1-acid-glycoprotein, α-2-macroglobulin, complement factor H, and others. These results point to the ubiquity and specificity of protein glycosylation alterations in HCC oncogenesis. It also confirms the robustness and validity of the targeted approach used in our study.

Increases in fucosylation, sialylation and branched structures observed in our studies are in line with earlier reports (22, 23, 46–48, 57); however, we could not identify identical glycopeptides across several NASH HCC analyses (21, 22, 31, 44, 57). A notable exception was a highly abundant biantenary disialylated glycan HexNAc(4)Hex(5)NeuAc(2) on position N207, which was previously shown to differentiate between early HCC, cirrhosis and healthy donors (31). There might be several reasons for this observed discrepancy: *(1)* preparation of samples involving deglycosylation and desialylation of haptoglobin; *(2)* targeted analysis of selected glycosylation sites, and *(3)* differences in etiologies of examined samples. While the most comprehensive glycosylation analyses are published on HCC with NASH etiology, our samples originated mainly from patients with viral hepatitis. It is known that both HCV and HBV not only employ N-glycosylated receptors and co-receptors for their attachment and entry into host cells (58), but that they also up-regulate expression of glycosyltransferases responsible for modifications of glycopeptide bonds (59). Analyses of the N-glycan profiles in the human hepatoma cell line Huh7.5.1 following HCV infection demonstrated enhanced levels of fucosylated, sialylated and complex N-glycans, with several glycan patterns overlapping with our results (60). Subtle differences in haptoglobin fucosylation patterns between HCC and liver cirrhosis HBV, HCV and ALC etiologies have been described previously by Zhu et al. (44); we hypothesize that these previously described glycosylation biomarkers might have been specific for non-viral etiology and/or influenced by sample preparation. Although the number of samples in our studies did not allow for in-depth analyses of each HCC etiology and stage, we observed stable diagnostic performance of the top glycopeptides in both cirrhotic HBV and HCV groups and cirrhotic- and non-cirrhotic patients from mixed etiologies (**Figure 5**). Due to the low number of samples no conclusions can be drawn regarding whether glycosylation changes might be specific to viral or non-viral HCC etiology.

To our knowledge, our study is the first to evaluate the differences between the AUC values of glycobiomarkers among different Hp phenotypes. We observed that the AUC of biomarkers only slightly depends on the Hp phenotype of the patient cohort. Previously, it has been reported that Hp phenotype distribution can vary considerably depending on the genomic distribution of Hp1 and Hp2 alleles in different ethnicities. For example, in Western Europe the majority of the population has type 2-1 Hp (41), whereas in South Asia type 2-2, and in some African countries, type 1-1 are the dominant phenotypes (61). It would be interesting to perform similar analyses in cohorts from other geographical regions for independent validation of results.

To date, the role of Hp glycosylation and its involvement in oncogenesis remains unclear. Different Hp types have different characteristics in binding to hemoglobin and activating the immune system (26, 62, 63). Moreover, we have previously observed that different Hp types, specifically type 1-1, have different glycosylation patterns and, even in a healthy population, Hp polymorphism affects its N-glycosylation pattern in plasma (64). An interesting hypothesis was raised by Wu et al. (2018) (65) in a study using a glycoproteomics approach to examine the impact of Hp glycosylation on its interactions with hemoglobin. Focusing on the Hp 1-1 phenotype, they demonstrated that N-glycan branching attenuates the affinity of the binding of Hp to dimeric oxygenated hemoglobin and, in contrast, fucosylation stabilizes protein binding. Tamara et al. (2020) (66) extended the studies to other Hp phenotypes and confirmed that Hp glycosylation status, its oligomeric state and proteolytic processing all correlate with the strength of Hp–hemoglobin binding. Moreover, as glycosylation of the proteins increases, their stability by protecting from proteolysis and degradation, glycosylated Hp can display an increased half-life compared with its average of 4 days (67–69). Altogether, these data suggest a scenario in which specifically glycosylated Hp with an extended half-life loses its efficiency to detoxify plasma from hemoglobin, leading to local oxidative stress and thereby promoting oncogenesis. During our study, we observed tetra-sialylated glycans to be increased both in in early- and late-stage HCC compared with controls. It has been shown that an increase of the highly sialylated glycans can negatively regulate the host anti-tumor immune response. It has also been reported that proteins with highly sialylated glycans have a masking effect for the tumor antigens and help them to escape the immune system (70–72). Although there is no direct evidence for Hp, an increase of highly sialylated glycans of Hp in early and late-stage of HCC may affect its immunogenicity.

In order to better understand the heterogeneity and stability of the identified Hp glycosylated variants, a dynamic approach under therapy may be used. This type of study would help determine whether the Hp glycosylation changes are dynamic and reversible, and if they have the potential to act as biomarkers to track the effect of therapy on patients.

Despite the encouraging results, we acknowledge some limitations in our study. First, although the approach we used is optimal for finding novel biomarkers with minimum pre-knowledge, we observed missing values for some of the identified glycopeptides in some of the samples in the cohort. Thus, the promising glycopeptides for the diagnosis of early-stage HCC identified in this study need to be validated in an independent cohort, using a targeted MS method to avoid missing values in the dataset. Increasing the number of patients for specific etiologies and stages would also allow in-depth analyses of the subtle differences of glycosylation patterns in viral and non-viral hepatitis, and HCC progression. Second, the analytical methods used in this study are more complex and time-consuming than the conventional antibody-based methods used for measurement of AFP. In the next steps of development to validate and design a diagnostic test, targeted LC-MS methods could be applied, which are less complex. Moreover, due to the high abundance of Hp in the serum and plasma, it might be feasible to skip the Hp immunoisolation step from the workflow. The methodology should be improved before implementation into clinical practice.

In conclusion, this study has revealed new glycobiomarkers with high diagnostic values for the early detection of HCC, with the approach applied in a relatively large cohort. These biomarkers may be used to differentiate early-stage HCC from various liver diseases, and thus could enable physicians to intervene with the first/early range of treatments, such as liver ablation, to increase the survival rate of patients with HCC. Moreover, the investigation proved the importance of studying glycan and glycopeptides for the discovery of novel biomarkers. A similar method could be used to discover glycobiomarkers for other major cancer types, such as ovarian, lung, breast and pancreatic cancers.

## Data availability statement

The original contributions presented in the study are included in the article/**Supplementary Material**, further inquiries can be directed to the corresponding author/s.

## Ethics statement

The studies involving humans were approved by The Independent Ethics Committee (IEC). The studies were conducted in accordance with the local legislation and institutional requirements. The participants provided their written informed consent to participate in this study.

## Author contributions

All authors contributed to the study conception and design. Material preparation, data collection and analysis were performed by MK-N, MSL and KK. The first draft of the manuscript was written by MK-N, and all authors commented on previous versions of the manuscript. All authors contributed to the article and approved the submitted version.
