# Supplementary material for: Discovery of a haptoglobin glycopeptides biomarker panel for early diagnosis of hepatocellular carcinoma
Source: Front Oncol. 2023 Oct 18;13:1213898. doi: 10.3389/fonc.2023.1213898 (PMC10619681; doi:10.3389/fonc.2023.1213898)
Supplement: Supplementary file 1 [file Table_1.docx]

# Discovery of a Haptoglobin Glycopeptides Biomarker Panel for Early Diagnosis of Hepatocellular Carcinoma: Supplementary Data

**Supplementary Table 1: Numbers of patients with different HCC etiologies**

| **Etiology** | **HCC (n)** | | | **Controls (n)** |
| --- | --- | --- | --- | --- |
|  | **Early** | **Late** | **All** | **All** |
| **Cirrhotic ASH** | 3 | 4 | 7 | 0 |
| **Cirrhotic HBV** | 14 | 14 | 28 | 12 |
| **Cirrhotic HCV** | 16 | 4 | 20 | 14 |
|  | | | | |
| **Cirrhosis** | 40 | 26 | 66 | 26 |
| **Non-cirrhosis** | 10 | 6 | 16 | 31 |

Due to mixed etiologies and comorbidities, the results do not match the results presented in Table 1.

ASH, alcoholic steatohepatitis; HBV, hepatitis B virus; HCC, hepatocellular carcinoma; HCV, hepatitis C virus.

**Supplementary Table 2: The effect of HCC etiologies on the diagnostic performance of glycobiomarkers**

|  | **AUC for early HCC (%)** | | | |
| --- | --- | --- | --- | --- |
|  | **Cirrhotic HBV (n=14)** | **Cirrhotic HCV (n=16)** | **Cirrhosis (all etiologies) (n=40)** | **Non-cirrhosis (all etiologies) (n=10)** |
| **HexNAc(6)Hex(7)Fuc(1)NeuAc(4)** | 86.3 (71.7,100) | 93.3 (81.8,100) | 92.5 (85.6,99.4) | 96.5 (89.3,100) |
| **HexNAc(2)Hex(8)** | 75 (54,96) | 74.6 (56.4,92.8) | 78.3 (66.5,90) | 87.4 (70.8,100) |
| **HexNAc(5)Hex(6)Fuc(1)NeuAc(2)** | 72.6 (52.6,92.6) | 74.6 (55.5,93.6) | 75.7 (63.8,87.5) | 92.3 (84,100) |
| **HexNAc(2)Hex(9)** | 79.2 (59.8,98.5) | 64.7 (44.4,85.1) | 74.4 (62.3,86.6) | 91.9 (82.5,100) |
| **AFP** | 68.5 (47.2,89.7) | 61.2 (40,82.3) | 63.2 (48.8,77.5) | 89.4 (76.9,100) |

Due to mixed etiologies and comorbidities, the results do not match the results presented in Table 1.

AFP, alpha-fetoprotein; AUC, area under the curve; Fuc, fucose; HBV, hepatitis B virus; HCC, hepatocellular carcinoma; HCV, hepatitis C virus; Hex, mannose (glucose, galactose); HexNAc, N-acetylglucos(galactose)amine; NeuAc, N-acetylneuraminic acid.

**
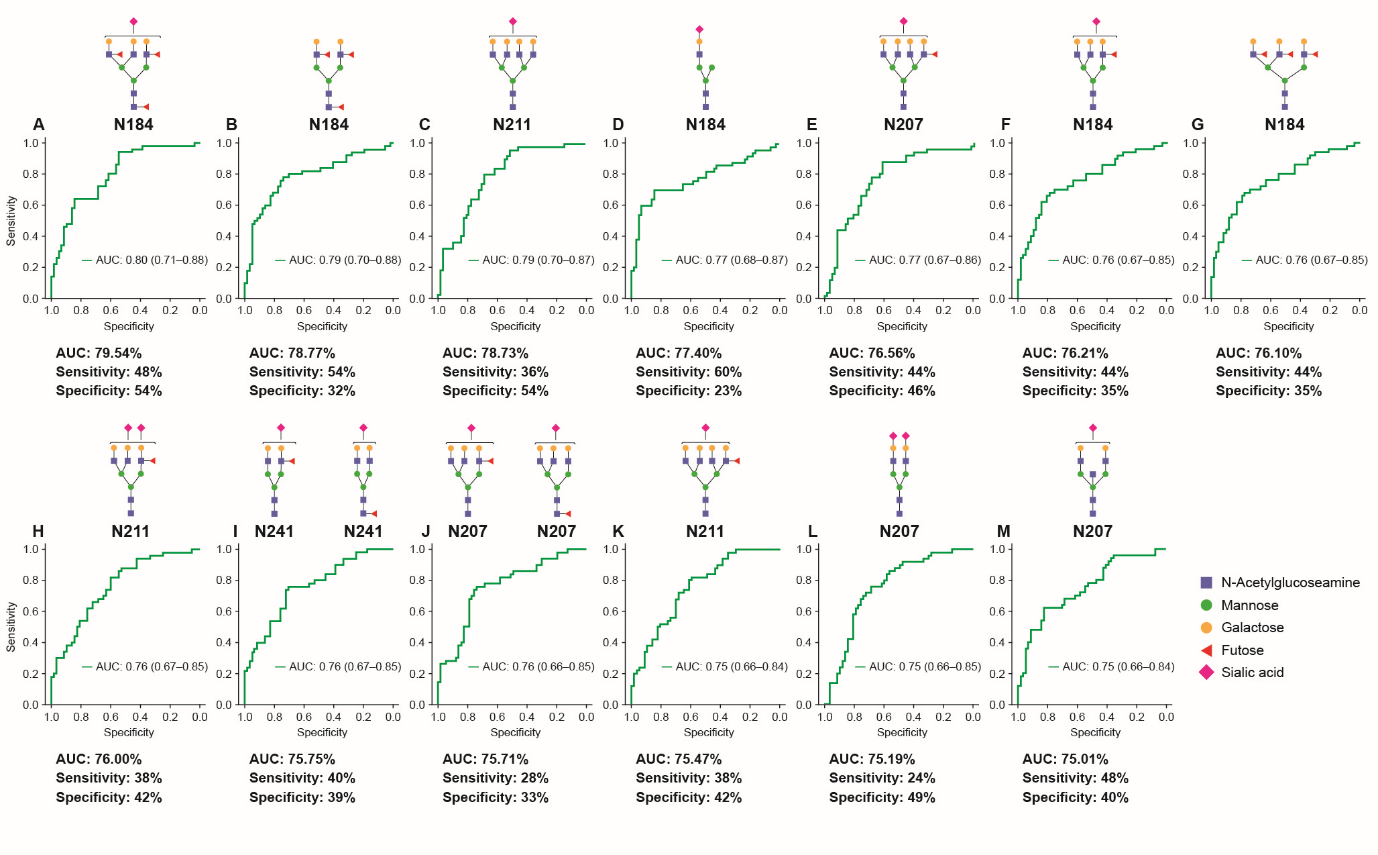
**

**Supplementary Figure 1: Clinical values of differentially regulated glycopeptides for diagnosis of early-stage HCC.**

The ROC curve of 13 glycopeptides (putative N-glycan structures are shown) with AUC between 75-80% for diagnosis of early-stage HCC are depicted. The specificity and sensitivity values are reported at fixed sensitivity and specificity cutoffs of 0.9, respectively.

AUC, area under curve; HCC hepatocellular carcinoma; ROC, receiver operating characteristic.


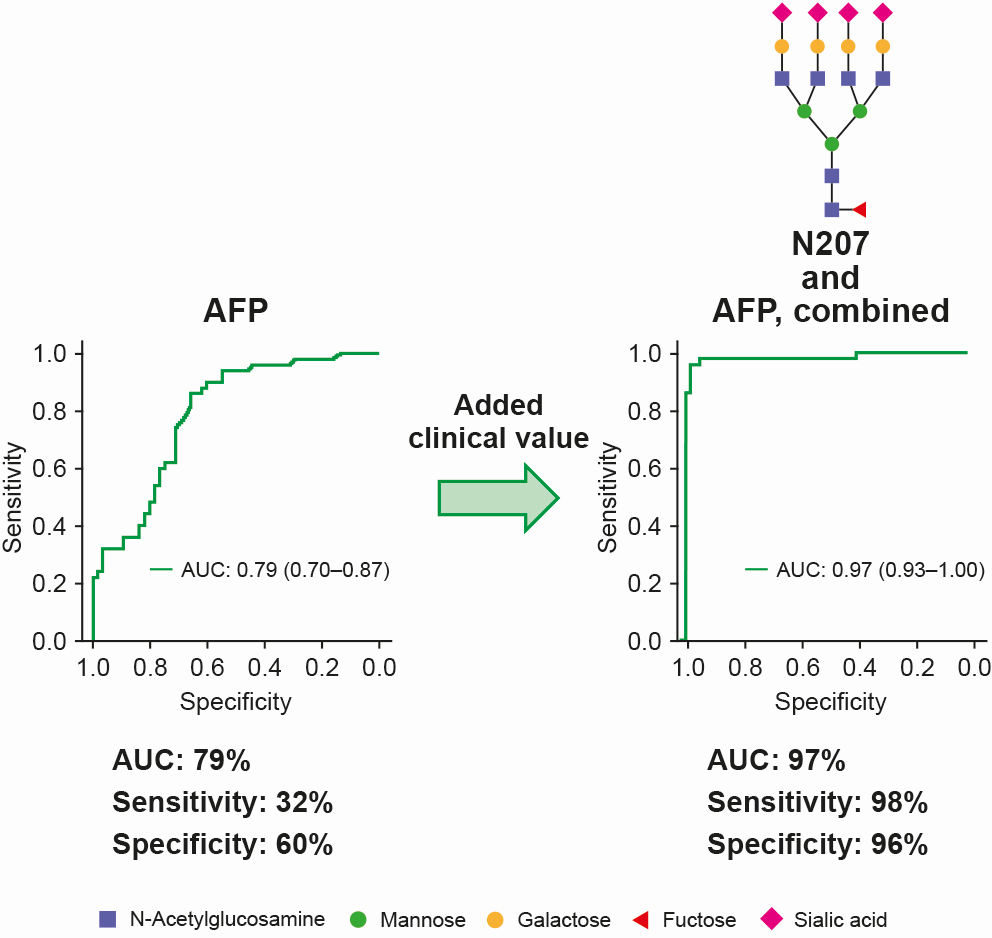


**Supplementary Figure 2: Combination with most significant glycopeptide in this study improves the clinical value of AFP.**

The AUC value for AFP for early diagnosis of HCC in our cohort is 79%. The most significant upregulated glycopeptide in our study can increase the AUC of AFP to 97%. The specificity and sensitivity values are reported at fixed sensitivity and specificity cutoffs of 0.9, respectively.

AUC, area under curve; AFP, alpha-fetoprotein; HCC, hepatocellular carcinoma.
